# Can nutrition prevent the onset of Rheumatoid Arthritis? A mini review of dietary risk and progression from pre-clinical autoimmunity

**DOI:** 10.3389/fnut.2026.1864271

**Published:** 2026-10-26

**Authors:** Maria V. M. Karadimova-Watts, Elena Philippou, Elena Nikiphorou

**Affiliations:** 1 GKT School of Medical Education, King's College London, London, United Kingdom; 2 Department of Life Sciences, School of Life and Health Sciences, University of Nicosia, Nicosia, Cyprus; 3 Department of Nutritional Sciences, King's College London, London, United Kingdom; 4 Centre for Rheumatic Diseases, School of Immunology and Microbial Sciences, Faculty of Life Sciences and Medicine, King’s College London, London, United Kingdom; 5 Centre for Education, Faculty of Life Sciences and Medicine, King’s College London, London, United Kingdom; 6 Department of Rheumatology, King's College Hospital, London, United Kingdom

**Keywords:** anti-inflammatory diet, autoimmune disease, gut microbiome, inflammation, Mediterranean diet, pre-clinical RA, Rheumatoid Arthritis

## Abstract

Rheumatic and musculoskeletal diseases are major causes of disability worldwide, and many have inflammation at their core. Rheumatoid Arthritis (RA), in particular, is preceded by a prolonged “pre-clinical” phase in which genetic susceptibility and modifiable exposures, including diet, interact. Nutrition is thought to play an important role as an ‘external’ but modifiable factor in inflammatory diseases. The existing literature suggests that it may act as a lever for prevention and risk modification through direct or indirect effects on adiposity, systemic inflammation, mucosal immunity, the gut microbiome, and cardiometabolic comorbidity. In this mini review, we synthesize and critically appraise the evidence linking dietary patterns and targeted nutritional interventions with RA risk.

## 1 Introduction

Rheumatoid Arthritis (RA) is a systemic autoimmune disease characterized by joint inflammation (1) and is estimated to have a global prevalence of 460 per 100,000 population (2). These inflammatory processes lead to joint and bone damage, pain, and stiffness (3). RA symptoms are progressive and vary among individuals; pain, stiffness, fatigue, and deformity can disrupt physical, psychological, and sexual functioning (4). Furthermore, the inflammatory effects of RA spread beyond the joints, increasing morbidity and mortality through complications in the heart, lungs, nervous system, and vascular system (5).

RA incidence and prevalence vary globally; a 2025 systematic review identified wide variation between regions and countries (6) with a prevalence of 541–779 per 100,000 in the UK (7), 680 per 100,000 in Canada (8) and 1,500 per 100,000 in Korea (9). These geographic variations may reflect ambiguous diagnostic criteria (10), and uncertainty due to insidious onset and gradual symptom progression (11). Many genetic, socioeconomic, and environmental factors have been identified as potential influences on RA prevalence (12). The complex interaction between genetic, hormonal, immune systems, and environmental factors on the expression of autoimmune disease has been referred to as the ‘mosaic of autoimmunity’ (13), whereby the above factors converge to produce unique rheumatological presentations.

Regarding environmental exposures, several studies have highlighted the beneficial effect of an anti-inflammatory diet in established disease on both RA-disease activity (14), and patient-reported pain (15). Chronic inflammatory states are thought to stem from hyperresponsivity to normally benign stimuli, loss of protective cellular barrier function, inflammatory cell infiltration, and overproduction of inflammatory mediators such as chemokines, cytokines, and oxidants (16). In this context, anti-inflammatory diets may decrease inflammatory cell production, promoting greater intestinal cell barrier function, and increasing anti-inflammatory responses (16). Characteristics of anti-inflammatory diets include being high in monounsaturated fats (for example, extra virgin olive oil; EVOO) and n-3 long-chain polyunsaturated fatty acids (PUFAs), while also being low in glycemic index, saturated fat, and added sugar and rich in fiber and phytonutrients, through a variety of fruits, nuts and seeds, vegetables, and legumes (17). An anti-inflammatory diet aligns closely with the Mediterranean diet (MD), and thus some researchers recommend that the MD be used alongside standard medical treatments in RA management (17–19).

Dietary intake influences an individual’s body mass index (BMI) and adiposity (20, 21). In turn, excess adipose tissue has been shown to harbor greater inflammatory cells (such as neutrophils, lymphocytes and macrophages), thus contributing to systemic inflammation (22). Macrophages, which are particularly abundant in adipose tissue, are responsible for the secretion of pro-inflammatory cytokines (22), of which tumor necrosis factor (TNF) and interleukin (IL) 1 and 6, are heavily implicated in RA (23). Strong associations have been drawn between adiposity and increased risk of developing RA (21), highlighting that nutrition may be implicated in disease development via increasing BMI.

Furthermore, bacterial colonization in the gut, particularly *Prevotella copri (Pc)* and *Collinsella aerofaciens,* may result in pro-inflammatory intestinal environments, a potential mechanism driving progression to RA (24). This suggests a direct link between diet, the microbiome, and RA risk. Additionally, nutrition may be associated with the development of metabolic comorbidities common in RA, such as several cardiovascular comorbidities, including myocardial infarction, congestive heart failure, and atrial fibrillation (25). Prioritizing diet in RA patients may therefore not only reduce inflammation and improve disease status, but also slow the progression of cardiometabolic comorbidities (26).

This mini review aims to synthesize and critically appraise the current evidence linking dietary patterns and targeted nutritional interventions with RA risk. To inform the review, a search was carried out in PubMed and Google Scholar using the following search terms: Rheumatoid Arthritis, Arthralgia, Diet, Nutrition, Mediterranean Diet, Western Diet, Anti-Inflammatory Diet and Pre-Clinical Rheumatoid Arthritis. Systematic reviews, observational studies and interventional studies were included based on their relevance to the development of RA.

## 2 Rheumatoid Arthritis risk and Mediterranean diet, Western diet, and overall dietary quality

A commonly studied dietary pattern in relation to RA risk is the MD, characterized by abundant plant foods such as unrefined cereals, fruit, vegetables, legumes, EVOO, moderate consumption of poultry, dairy products, and eggs, and limited consumption of sweets and red meat (17). In the French E3N-EPIC prospective cohort study in women (1990–2017), adherence to a MD in those with established smoking history was associated with a lower risk of RA (HR 0.91 [95% CI 0.84–0.99]) (27). No association, however, was seen in non-smoking women. This highlights the effect of environmental influences on RA risk, such as smoking, as demonstrated by a dose–response meta-analysis of 10 studies, showing that the risk of RA in those with a 21–30 pack-year smoking history, compared to never-smokers, was increased by 94% (RR 1.94 [95% CI 1.65–2.27]) (28). The association between greater adherence to the MD and reduced RA risk, in both men and women, has also been supported by evidence from a cohort study and systematic review including 117,341 participants, where those in the highest quartile of MD adherence had a lower risk of RA development than those in the lowest quartile (HR 0.71 [95% CI 0.58–0.88]) (29). This association appeared to differ according to seropositive status. Johansson et al. (30) reported that greater adherence to the MD was associated with a reduced risk of RF-positive RA (OR 0.69, 95% CI 0.54–0.88), whereas no significant association was observed for RF-negative RA.

In contrast the ‘Western Diet’ (WD) - characterized by high intakes of red and processed meat, refined carbohydrates, added sugars, high salt, and ultra-processed foods (UPFs) - has been proposed as a potential driver of autoimmune disease (19). Red meat may contribute to a pro-inflammatory dietary pattern, partly through arachidonic acid (31). UPFs are industrial formulations with five or more ingredients (32), and additives such as preservatives, emulsifiers, sweeteners, and artificial colors/flavors (33). Examples include breakfast cereals, processed meats, soft drinks, and pre-packaged frozen meals (34). Although most foods undergo some processing, additives dictate the degree of processing: standard steel-cut oats are minimally processed, become processed when they contain sugar, and ultra-processed through the addition of flavors/colorings (35). The low fiber content of the WD is associated with fewer fiber-degrading bacteria and increased expression of virulence factors (36), reducing microbial diversity and depriving beneficial gut bacteria of fermentable substrates. This reduces production of protective short-chain fatty acids such as butyrate which regulate immune cells (37). Reduced butyrate may increase intestinal permeability, facilitating microbial product translocation into the circulation and promoting inflammation (38).

High consumption of UPFs is also associated with a higher obesity prevalence (36) and increased RA risk. In 207,012 participants of the UK Biobank study who were free from RA at baseline, high consumption of UPFs was associated with a 6% increase in RA risk per standard deviation increase in UPF intake (HR 1.06 [95% CI 1.01–1.11]) (39). UPFs are thought to contribute to systemic inflammation, leading to a rise in inflammatory markers such as C-reactive protein (CRP) and leucocytes (39).

Conversely, better diet quality is associated with lower RA risk. In a prospective study of 170,000 women from the Nurses’ Health Study who were free from RA at baseline, greater long-term adherence to the 2010 Alternative Healthy Eating Index (AHEI-2010) was associated with a lower risk of seropositive RA among women aged ≤55 years (HR 0.67 [95% CI 0.51–0.88]) (40). Similarly Di Giuseppe et al. (41) prospectively followed 32,232 women for 7.5 years, as part of the Swedish Mammography Cohort. Findings included a non-statistically significant 29% (RR 0.71 [95% CI 0.48–1.04]) reduced RA risk in women who consistently ate ≥1 serving of fish per week and a 52% (RR 0.48 [95% CI 0.29–0.67]) lower risk in those who consumed >0.21 g/day of n-3 PUFAs. Omega-3 fatty acids [synonymous with n-3 PUFAs (42)], found in fatty fish, seafood, walnuts, and linseeds (43), are an essential fatty acid component of the human diet. They exert anti-inflammatory effects partly by competing with arachidonic acid for cyclooxygenase enzymes, thereby reducing the production of pro-inflammatory arachidonic acid-derived prostaglandin E2 and favoring formation of the less inflammatory prostaglandin E3 (44).

## 3 Progression from pre-clinical autoimmunity to clinically apparent disease

### 3.1 Pre-clinical Rheumatoid Arthritis and risk

Pre-clinical RA is the period preceding the clinical phase of RA by several years (45) during which initial inflammatory processes and sub-clinical synovial inflammation may develop before symptom manifestation. Patients may exhibit anti-citrullinated protein antibodies (ACPAs) or RF positivity during this phase (3). Notably, some patients only develop ACPAs after the onset of clinically-apparent RA, therefore, ACPAs cannot reliably be used as a biomarker of pre-clinical RA (46). The pre-clinical phase is thought to be influenced by genetic and environmental factors: non-modifiable genetic factors contributing approximately 30–60% of susceptibility to RA development (46), with the remainder attributed to environmental factors (47). High decaffeinated coffee consumption, smoking history, and exposure to air pollution have been associated with increased susceptibility to RA, potentially through gene–environment interactions that promote the development of autoimmunity (46). Lifestyle modification (including diet alteration to favor anti-inflammatory diets such as the MD) has been associated with a lower risk of RA development (45), potentially modulating the gene–environment interaction (48).

### 3.2 Dietary links and immune activation

Anti-inflammatory diets such as the MD may reduce inflammation through cytokine regulation by lowering the production of pro-inflammatory molecules, such as IL-6 and oxidative stress markers (49). Furthermore, n-3 PUFAs, key components of the MD, may counteract the secretion of pro-inflammatory IL-6 in vascular tissues, exerting anti-inflammatory effects (50). Some evidence for this stems from a nested case–control study in a population without RA but at high genetic risk, where anti-CCP2 positivity was inversely associated with n-3 PUFA concentrations in erythrocytes (OR 0.47 [95% CI 0.24–0.92] per standard deviation increase) (51). It is of note, however, that subsequent progression to clinical RA was not studied.

Beyond these systemic anti-inflammatory effects, diet may influence immune processes at mucosal surfaces. The mucosal origins hypothesis provides a conceptual rationale for the potential role of these effects in RA initiation. The hypothesis argues that immune dysregulation at mucosal sites may contribute to systemic autoimmunity that precedes clinically apparent synovitis (52). Mucosal immunity is promoted through ingestion of soluble fibers from fruit, vegetables, and legumes, which nurture the growth of beneficial gut bacteria, protecting the intestinal barrier and reducing systemic inflammation (49). In contrast, WDs are thought to disrupt microbial diversity and increase expression of microbial virulence factors (36). They may also promote inflammation through diet-induced obesity, whereby the accumulation of white adipose tissue contributes to a pro-inflammatory state through its endocrine activity, including the production of TNF-α and IL-6, and modulation of T-cell responses (53).

Furthermore, WD constituents may directly trigger localized intestinal inflammation (through factors such as oxidized LDL cholesterol and saturated fatty acids), alongside indirectly triggering inflammation through microbial alteration (54). WDs can contribute to dysbiosis as they typically contain a higher concentration of UPFs and inflammatory foods (55). In particular, WDs are rich in additives (such as salt and artificial sweeteners), which have been associated with reduced numbers of beneficial gut bacteria *Lactobacillus* and *Bifidobacteria* (55). As such, gut dysbiosis may be promoted through the loss of beneficial organisms, growth of harmful bacteria and overall reduction in microbial diversity (56). Such dysbiosis may shift immune responses toward inflammatory pathways, including Th17 responses (52), and may facilitate abnormal modification of proteins such as citrullination (57). Given the physiological homeostasis between the gut microbiome and the immune system (56), pro-inflammatory diets which adversely alter microbial composition may also contribute to immune dysregulation. For example, *Pc* has been proposed as a potential contributor to the progression of pre-clinical to established RA, evidenced by significant abundance of *Prevotella* species (*p* = 0.04) in individuals with pre-clinical RA compared to first-degree relative controls (58), and anti-CCP-positive individuals at risk of RA showing a trend toward higher IgG anti-*Pc*-p27 levels (59).

Metabolic syndrome and obesity also contribute to chronic inflammatory states through increased oxidative stress, circadian rhythm disruption (60) and inappropriate activation of immune cells (61) such as T cells (53). Pathways described in Figure 1^1^ - The Role of Diet in the Onset of Rheumatoid Arthritis, suggest that diet could influence RA development and is a plausible component of a wider gene–environment interaction in pre-clinical RA. It is of note, however, that evidence remains limited.

**Figure 1:**
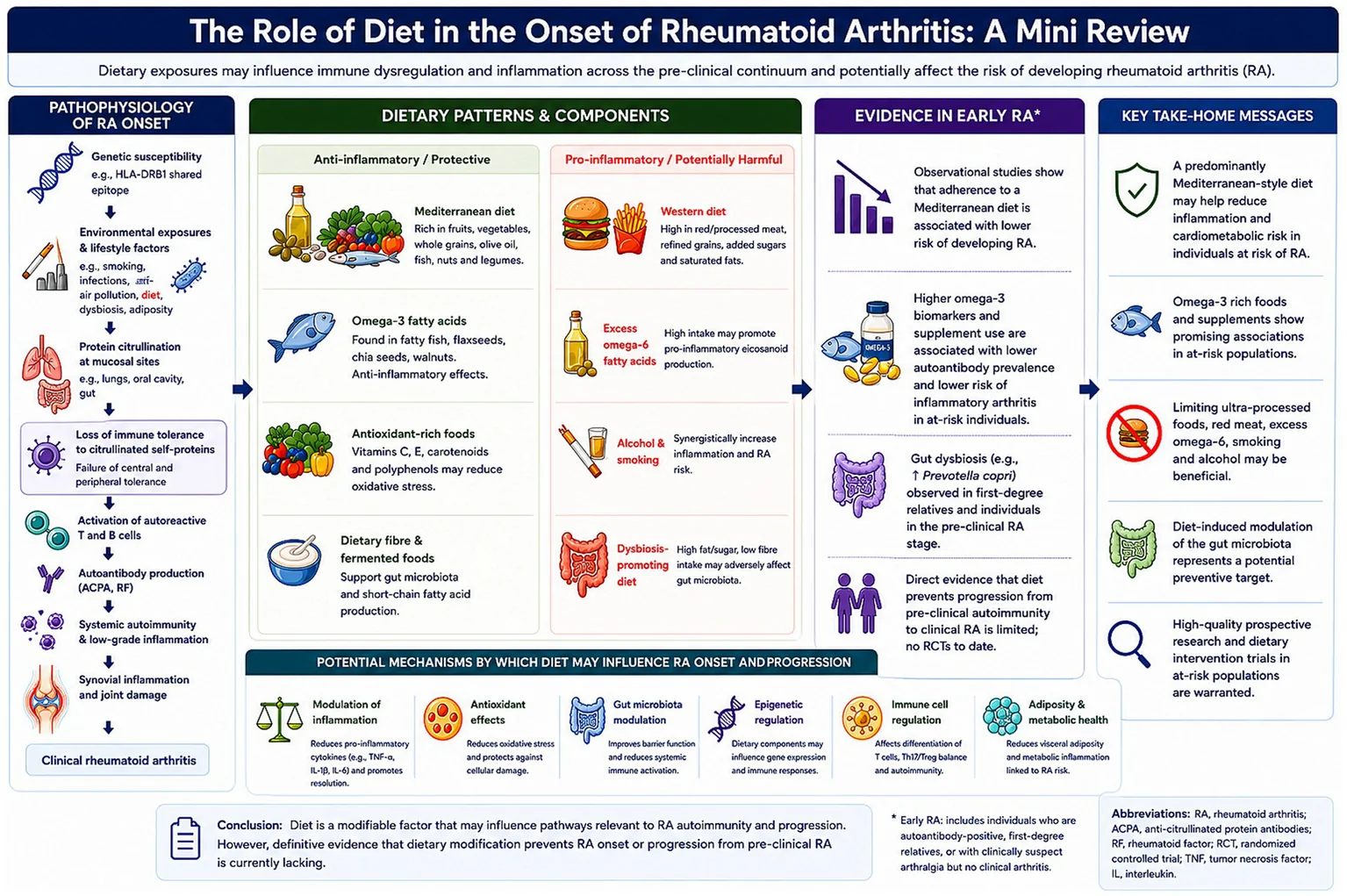
The role of diet in the onset of Rheumatoid Arthritis. RA, Rheumatoid Arthritis; ACPA, anti-citrullinated protein antibodies; RF, rheumatoid factor; RCT, randomized controlled trial; TNF, tumor necrosis factor; IL, interleukin.

## 4 Discussion

Cumulative evidence suggests that diet is important in the development of RA through a variety of anti-inflammatory and pro-inflammatory mechanisms. The available observational evidence generally suggests an association between greater adherence to the MD and lower RA risk (27, 29, 30, 41) exerted through anti-inflammatory components such as n-3 PUFAs (41, 50, 62) and plant-based foods and fiber promoting the growth of beneficial microbial bacteria (49). On the contrary, the WD is typically associated with pro-inflammatory changes (19, 36, 39, 53, 54, 56). In genetically susceptible people, particularly in the presence of other exposures such as smoking or obesity (63), these inflammatory changes may promote autoantibody formation, years before apparent joint swelling.

Notably most evidence reviewed is derived from studies on established RA, rather than pre-clinical and at-risk individuals, and direct evidence that dietary modification can alter the progression to clinical RA remains limited. With this caveat in mind, several confounding factors, including smoking, may alter the effect of diet on the pre-clinical RA course (27). It is thus crucial to acknowledge the concept of the mosaic of autoimmunity when considering modifiable risk factors for RA, as some factors may prove challenging to separate. Another important consideration is adherence to the MD (29, 30), which can be challenging to assess across different populations and study designs, thereby limiting comparability between studies. Moreover, although evidence on diet in pre-clinical RA is largely observational, evidence on the impact of diet on established RA may still provide useful mechanistic and clinical insights. For example, a systematic review of clinical trials evaluating dietary interventions in RA found that their effects were more notable for subjective rather than objective measures of RA disease activity (64). This provides foundational evidence that dietary interventions can play a role in RA; however, there remains scope to focus more specifically on how diet can influence the pre-clinical RA course, rather than established disease. Identifying pre-clinical RA can be challenging due to the variety of causes of arthralgia and the lack of universal, highly sensitive serological markers. Although RF and ACPA positivity may provide insight into a patient’s pre-clinical RA phase, not all patients who develop RA will exhibit these autoantibodies during this period (46). This is important given that higher MD adherence in pre-clinical RA patients with RF positivity was associated with 31% lower odds of RA development (OR 0.69 [95% CI 0.54–0.88]) (30).

An additional point to consider is the effect of other well-known diets on RA risk, although available evidence is primarily based on established rather than pre-clinical RA. The ketogenic diet has been reported to have anti-inflammatory effects in established RA through suppression of IL-1 and TNF-α (65) and vegan diets have also been associated with symptom reduction in established RA, likely due to the antioxidant effects and favoring the growth of *Lactobacillus* (66). These foundations provide evidence for the involvement of diet in RA disease activity, although further research is needed to assess the impact of a variety of diets on the pre-clinical RA disease course.

Despite cumulative evidence suggesting that anti-inflammatory diets may modulate RA outcomes, understanding patient and clinician perspectives is important for implementing dietary advice into current medical practice. Through surveying 100 individuals at risk of RA (who reported arthralgia and were seropositive for RF/ACPAs), Boheemen et al. (67) found that all patients were willing to change at least 1 lifestyle component: mainly opting to increase fruit and vegetable intake. Rheumatologists (*n* = 49) however, were not issuing lifestyle counseling to all at-risk RA patients, due to a perceived shallow supporting evidence base. Notably, the small sample sizes limit generalizability of findings.

### 4.1 Limitations and clinical implications

Evidence on RA risk relies heavily on observational studies, which cannot establish causality between diet and symptom burden. Furthermore, assessing dietary quality can be challenging, as the use of different dietary indices, such as MD adherence or the AHEI-2010, may reduce comparability between studies. Additionally, as pre-clinical RA cannot be reliably diagnosed, existing studies have not specifically recruited patients with pre-clinical RA but rather at-risk or general populations who were followed up to incident RA diagnosis. This limits the conclusions that can be drawn regarding the pre-clinical-to-clinical RA transition.

Nevertheless, growing evidence suggests that adoption of an anti-inflammatory MD may improve RA disease activity and reduce inflammation (17). Thus, the potential implications of diet on pre-clinical RA are considerable. Such a dietary pattern emphasizes plant-based foods including legumes, a variety of vegetables, whole grains, unsalted nuts and seeds, seasonal fruit (especially those rich in phytochemicals, such as berries), n-3 PUFAs (such as fatty fish), lean protein sources, moderate consumption of eggs and dairy products, fermented foods, and limited, if at all, consumption of red meat and UPFs. By targeting modifiable dietary risk factors in the pre-clinical RA phase, through, if necessary, individualized dietary advice provided by registered dietitians, it may be possible to alter the course of progression to clinical RA (48). However, further research is necessary to better understand which dietary interventions are most beneficial, the time-period of implementation and the population group to be targeted. A close collaboration between rheumatologists and registered dietitians is also important.

## 5 Conclusion

In conclusion, evidence suggests that dietary modification toward a Mediterranean dietary pattern may exert anti-inflammatory effects and beneficially influence the gut microbiome. In contrast, the WD is perceived to pose an environmental risk factor, either directly through inflammation or indirectly through its association with a higher risk of obesity, potentially increasing the risk of RA and co-morbidities. However, definitive evidence that adopting a MD alters progression from pre-clinical autoimmunity to clinical disease, is still lacking. Existing evidence is primarily observational and derived from general-population cohorts or individuals with established RA, rather than confirmed at-risk individuals. Future research should continue to evaluate the effects of specific anti-inflammatory dietary interventions during the pre-clinical phase of RA using robust, rigorously designed methodologies.

## Footnotes

1 Although several associations have been described as illustrated by Figure 1, many require further confirmation.
