# Supplementary material for: Can nutrition prevent the onset of Rheumatoid Arthritis? A mini review of dietary risk and progression from pre-clinical autoimmunity
Source: Front Nutr. 2026 Oct 26;13:1864271. doi: 10.3389/fnut.2026.1864271 (PMC13746278; doi:10.3389/fnut.2026.1864271)
Supplement: Supplementary file 1 [file Supplementary_file_1.docx]

Supplementary Material

**Use of Generative AI- Supplementary Information**

| Date | Input/ Prompt | Output |
| --- | --- | --- |
| 8/3/26 (ChatGPT) | Hi, please can you make a plan for a mansucript on 'Can nutrition prevent the onset of rheumatic inflammatory diseases? A mini review with a focus on Rheumatoid Arthritis' which will be 3000 words long. Please can you include the following headings: introduction, Incident Rheumatoid Arthritis and Related Inflammatory Disease Risk, Progression from Pre-clinical Autoimmunity to Clinically Apparent Disease, Discussion. Please can you list the suggested word count per section, and suggest some references that can be expanded upon in each section. Please ensure that the references are published only and from trustworthy sources. | Below is a **structured manuscript plan for a 3,000-word mini-review** titled: **“Can nutrition prevent the onset of rheumatic inflammatory diseases? A mini review with a focus on Rheumatoid Arthritis.”** The outline includes **suggested word counts, key themes to cover, and examples of reliable published references** (peer-reviewed journals or reputable organisations) that could be expanded upon in each section. - **Manuscript Plan (3000 words)** - **1. Introduction (≈500 words)** - **Key aims** - Introduce **rheumatoid arthritis (RA)** as a chronic systemic autoimmune disease characterised by synovial inflammation and progressive joint destruction. - Highlight the **multifactorial aetiology**: genetics (e.g., HLA-DRB1 shared epitope), environmental exposures (smoking), microbiome, and lifestyle factors. - Introduce **nutrition as a modifiable environmental factor** potentially influencing immune regulation and inflammation. - Emphasise the concept of **pre-clinical RA**, where immune dysregulation precedes symptoms by years. - Frame the **central question**: whether diet could prevent disease onset or modify progression from risk states. - Briefly outline the scope of the review: focusing primarily on RA but referencing other inflammatory rheumatic diseases when relevant. - **Key concepts to include** - Inflammatory and immune-modulating effects of diet - The “Western diet” vs anti-inflammatory dietary patterns - Interaction with obesity, microbiome and metabolic inflammation - The growing interest in **dietary prevention strategies** - **Suggested references** - Nikiphorou & Philippou, *Autoimmunity Reviews*, 2023 – overview of nutrition and RA risk/management. ([PubMed](https://pubmed.ncbi.nlm.nih.gov/37182439/?utm_source=chatgpt.com)) - Hu et al., *Nature Reviews Rheumatology*, 2021 – environmental risk factors in RA. - Smolen et al., *The Lancet*, 2016 – RA pathogenesis and epidemiology. - Calder et al., *British Journal of Nutrition*, 2020 – diet and inflammation. - Deane et al., *Annals of the Rheumatic Diseases*, 2017 – preclinical RA concept. - **2. Incident Rheumatoid Arthritis and Related Inflammatory Disease Risk (≈900 words)** - **Purpose** To review **epidemiological evidence linking diet or nutritional factors with the development of RA and other inflammatory rheumatic diseases**. - **Subsections to consider** - **2.1 Dietary patterns and RA risk** - Mediterranean diet - Western diet - Dietary inflammatory index Evidence suggests adherence to a Mediterranean dietary pattern may be associated with lower RA risk in observational cohorts. ([PubMed](https://pubmed.ncbi.nlm.nih.gov/32909390/?utm_source=chatgpt.com)) - **2.2 Specific nutrients and food groups** Discuss evidence for: **Potential protective factors** - Omega-3 fatty acids (fish consumption) - Fruits, vegetables, antioxidants - Fibre and microbiome effects - Vitamin D Higher fatty-fish intake has been associated with a reduced risk of developing RA in prospective cohorts. ([PubMed](https://pubmed.ncbi.nlm.nih.gov/15996059/?utm_source=chatgpt.com)) **Potential risk factors** - Ultra-processed foods - High red-meat consumption - Diets high in saturated fat and refined carbohydrates Higher consumption of ultra-processed foods has been associated with increased RA risk in large cohort analyses. ([UK Biobank](https://www.ukbiobank.ac.uk/publications/association-of-ultra-processed-food-consumption-with-risk-of-rheumatoid-arthritis-a-retrospective-cohort-study-in-the-uk-biobank/?utm_source=chatgpt.com)) - **2.3 Evidence from cohort and case-control studies** - Nurses’ Health Study - E3N-EPIC cohort - UK Biobank analyses These studies highlight that **dietary patterns may influence RA susceptibility**, although results remain heterogeneous. - **2.4 Evidence in other inflammatory rheumatic diseases** - Psoriatic arthritis - Systemic lupus erythematosus - Ankylosing spondylitis Focus on shared inflammatory pathways and metabolic factors. - **Suggested references** Key papers to expand on: - Nguyen et al., *Arthritis & Rheumatology*, 2021 – Mediterranean diet and RA risk. ([PubMed](https://pubmed.ncbi.nlm.nih.gov/32909390/?utm_source=chatgpt.com)) - Hu et al., *Annals of the Rheumatic Diseases*, 2017 – diet and RA risk in Nurses’ Health Study. - Zhao et al., *American Journal of Clinical Nutrition*, 2024 – ultra-processed foods and RA risk. ([UK Biobank](https://www.ukbiobank.ac.uk/publications/association-of-ultra-processed-food-consumption-with-risk-of-rheumatoid-arthritis-a-retrospective-cohort-study-in-the-uk-biobank/?utm_source=chatgpt.com)) - Tedeschi et al., *Arthritis Care & Research*, 2017 – dietary quality and RA risk. - Sparks et al., *Current Rheumatology Reports*, 2018 – lifestyle factors and RA development. - **3. Progression from Pre-clinical Autoimmunity to Clinically Apparent Disease (≈900 words)** - **Purpose** To discuss how **nutrition might influence the transition from preclinical autoimmunity to symptomatic inflammatory arthritis**. - **Subsections** - **3.1 Preclinical RA and risk states** - Presence of autoantibodies (ACPA, RF) - Arthralgia and subclinical inflammation - Genetic susceptibility Key concept: **immune dysregulation precedes symptoms by years**. - **3.2 Mechanisms linking diet and immune activation** Potential biological pathways: **Inflammatory modulation** - Dietary fatty acids and cytokine production - Oxidative stress and antioxidant intake **Gut microbiome** - Diet-induced microbiota changes - Microbial metabolites and immune signalling Changes in gut microbial metabolites may influence immune activation and bone erosion in RA. ([arXiv](https://arxiv.org/abs/2307.08848?utm_source=chatgpt.com)) **Metabolic pathways** - Obesity-related inflammation - Insulin resistance - Adipokines - **3.3 Diet and biomarkers of RA risk** Discuss studies linking diet to: - C-reactive protein - Cytokines - Autoantibody formation - Dietary inflammatory index - **3.4 Interventional evidence in high-risk populations** Evidence is limited but emerging: - Omega-3 supplementation - Mediterranean diet trials - Microbiome-targeted nutrition Highlight the **lack of large RCTs in pre-clinical RA populations**. - **Suggested references** - Deane et al., *Annals of the Rheumatic Diseases*, 2017 – prevention strategies in RA. - Sparks, *Rheumatic Disease Clinics of North America*, 2018 – lifestyle risk factors. - Nikiphorou & Philippou, *Autoimmunity Reviews*, 2023 – diet and immune pathways in RA. ([PubMed](https://pubmed.ncbi.nlm.nih.gov/37182439/?utm_source=chatgpt.com)) - Scher et al., *Nature Reviews Rheumatology*, 2016 – microbiome and RA. - Rosser & Mauri, *Nature Reviews Immunology*, 2018 – regulatory immune pathways. - **4. Discussion (≈700 words)** - **Key themes** - **4.1 Strength of current evidence** - Observational data suggests **diet may influence RA risk**, but causality remains uncertain. - Evidence strongest for: - Mediterranean-style diets - Omega-3 intake - Reduced ultra-processed foods. - **4.2 Limitations of the literature** - Heterogeneity of dietary measurement methods - Confounding lifestyle factors - Reverse causality in case-control studies - Limited intervention trials - **4.3 Implications for prevention** Potential future strategies: - Dietary risk stratification - Nutritional counselling in high-risk populations - Integration with lifestyle prevention (smoking cessation, weight control) - **4.4 Future research directions** - Prospective cohorts with repeated dietary measures - Randomised trials in **individuals with preclinical RA** - Multi-omics studies (microbiome, metabolomics) - Precision nutrition approaches - **4.5 Clinical relevance** Even if prevention remains uncertain, dietary modification may still: - reduce systemic inflammation - improve comorbidities - support overall disease management - **Summary of Word Counts** **Section** \| **Word count**; Introduction \| 500; Incident RA and related disease risk \| 900; Progression from preclinical autoimmunity \| 900; Discussion \| 700; **Total** \| **3000 words** ✅ If helpful, I can also: - Draft a **high-quality first paragraph for the introduction** - Provide **20–25 key references formatted in Vancouver style** (useful for submission) - Help structure **figures or tables** (e.g., “Dietary factors associated with RA risk”). |
| 24/4/26 (Claude.AI) | Hello, please can you adjust/ refine/ develop a new figure which represents the same information as this current figure? Please make it suitable quality for publication in a journal, ideally with a width of 180mm, with a resolution of 300 dpi. |  |
